# Investigation on the Neural Mechanism of Hypnosis-Based Respiratory Control Using Functional MRI

**DOI:** 10.1155/2018/8182542

**Published:** 2018-07-02

**Authors:** Yanjun Liu, Wenjian Qin, Rongmao Li, Shaode Yu, Yini He, Yaoqin Xie

**Affiliations:** ^1^Institute of Biomedical and Health Engineering, Shenzhen Institutes of Advanced Technology, Chinese Academy of Sciences, Shenzhen 518055, China; ^2^Shenzhen Deep Bay Innovation Co., Ltd., Shenzhen 518055, China; ^3^Shenzhen College of Advanced Technology, University of Chinese Academy of Sciences, Shenzhen 518055, China; ^4^Key Laboratory for NeuroInformation of Ministry of Education, School of Life Science and Technology, University of Electronic Science and Technology of China, Chengdu 610054, China

## Abstract

Respiratory control is essential for treatment effect of radiotherapy due to the high dose, especially for thoracic-abdomen tumor, such as lung and liver tumors. As a noninvasive and comfortable way of respiratory control, hypnosis has been proven effective as a psychological technology in clinical therapy. In this study, the neural control mechanism of hypnosis for respiration was investigated by using functional magnetic resonance imaging (fMRI). Altered spontaneous brain activity as well as neural correlation of respiratory motion was detected for eight healthy subjects in normal state (NS) and hypnosis state (HS) guided by a hypnotist. Reduced respiratory amplitude was observed in HS (mean ± SD: 14.23 ± 3.40 mm in NS, 12.79 ± 2.49 mm in HS, *p*=0.0350), with mean amplitude deduction of 9.2%. Interstate difference of neural activity showed activations in the visual cortex and cerebellum, while deactivations in the prefrontal cortex and precuneus/posterior cingulate cortex (PCu/PCC) in HS. Within these regions, negative correlations of neural activity and respiratory motion were observed in visual cortex in HS. Moreover, in HS, voxel-wise neural correlations of respiratory amplitude demonstrated positive correlations in cerebellum anterior lobe and insula, while negative correlations were shown in the prefrontal cortex and sensorimotor area. These findings reveal the involvement of cognitive, executive control, and sensorimotor processing in the control mechanisms of hypnosis for respiration, and shed new light on hypnosis performance in interaction of psychology, physiology, and cognitive neuroscience.

## 1. Introduction

Respiratory control is one of the most essential parts for dose distribution management during radiotherapy, especially for lung and liver tumors. Conventional technologies of respiration control during radiotherapy include stable-respirationtraining before treatment, gating technology that coincides with the treatment in breath cycle [1], assistant visual system by showing standard respiration waveform to guide patients to breathe regularly, and real-time tumor tracking by implanting metallic or radio frequency fiducials [2,3]. These methods may prolong treatment time for gating, feeluncomfortable for patient, and even cause potential complications. In this study, hypnosis is introduced for respiratory control during radiotherapy without any side effects.

Many clinical evidences have proven that hypnosis is effective and safe in pain reduction [4], emotional stress reduction [4,5], which can be applied for treating depression [6], sleeping disorders [7], and anxiety [8], and other psychological therapy. Relative studies using electroencephalogram (EEG) [9,10] and functional magnetic resonance imaging (fMRI) [11–16] demonstrate the existence of neural brain activity in response to hypnotic suggestion. Additionally, respiration works in its neural regulation. Tiny variation in respiration (breathing rate or depth) and breath-holding attribute to the change of arterial level of carbon dioxide (CO_2_) therefore leading to increased cerebral blood flow (CBF) and blood oxygen level-dependent (BOLD) signal [17–21]. Similarly, an fMRI study of hyperventilation suggested that changed level of arterial CO_2_ comes with the BOLD signal [22]. Conversely, the chemoreflex triggered by the changing concentration of CO_2_ has an influence on respiratory variability (changing the breathing rate and depth) in return [23,24]. It forms a chemoreflex-mediated feedback cycle among respiration, CO_2_, CBF, and BOLD signal [20]. These studies provide the basis for investigation of neural mechanism of respiration control during radiotherapy.

As we know, lower amplitude of respiration is helpful for precise dose delivery, which can increase dose rate for tumor target, while protecting the organ at risk (OAR) from dose radiation. Our previous study has demonstrated that hypnosis can effectively reduce respiration amplitude and increase respiration stability [25]. In this study, we furthermore explore the mechanisms of hypnosis for respiratory control by using resting-state fMRI. The temporal variation [26] and signal synchronization [27,28] of BOLD signal were detected to investigate the correlative relationship between neural activity and respiratory motion.

## 2. Materials and Methods

### 2.1. Experimental Design

A wide distribution of physiological difference of eight volunteers (Table1) without history of neurological disorder participated in the hypnosis experiment. Intrasubject design was used, which consisted of two sections for every volunteer, corresponding to normal state (NS) and hypnosis state (HS), respectively. In the NS section, the volunteers were lying quietly in MRI, stayed awake with eyes closed and without any thinking activities. The NS section lasted about 10 minutes. In the HS section, the volunteers were guided into hypnosis by hypnotists to lead them into psychologically stable and comfortable state. The period of HS section lasted about 30–40 minutes. During both sections, the following three images for every subject were scanned: the structural coronal section of thoracic-abdomen, BOLD functional image, and structural T1 image of the brain. The structural thoracic-abdomen images were applied for analysis of respiratory motion, and the brain images (BOLD-fMRI and T1) were applied for analysis of spontaneous brain activities during hypnosis. It is noted that all of the volunteers are hypnotists themselves, and all of them are suitable for hypnosis.

#### 2.1.1. Ethical Statement

All methods were carried out in accordance with relevant guidelines and regulations. The experiment was approved by the Institutional Review Board of Shenzhen Institutes of Advanced Technology, Chinese Academy of Sciences. The informed consent was written in an approval document. Informed consent of the experiment was obtained from all subjects. Informed consent for publication of identifying information/images in an online open-access publication was obtained from all subjects.

### 2.2. Data Acquisition and Preprocessing

All of the data were acquired through a 3.0T SIEMENS MRI machine system. The scanning settings were as follows. Structural thoracic-abdomenparameters: repetition time and echo time (TR/TE) = 4.25/1.97 ms, slice thickness = 5 mm, flip angle (FA) = 30°, field of view (FOV) = 350 mm × 350 mm, matrix = 128 × 128, and frequency = 3 Hz. Functional image-scanning parameters: TR/TE = 2000/30 ms, slice thickness = 4 mm, FA = 90°, FOV = 220 mm × 220 mm, and matrix = 64 × 64. T1 image-scanning parameters: TR/TE = 2000/9.2 ms, slice thickness = 4 mm, FA = 130°, FOV = 230 mm × 130 mm, and matrix = 320 × 182.

The functional image processing was performed by RESTplus_V1.2 (www.restfmri.net), SPM8 (www.fil.ion.ucl.ac.uk/spm), and data analysis toolkits for resting state fMRI, running on MATLAB platform. The preprocessing procedures included time points removal (the first five time points were removed to avoid the unstable operation in the beginning of scan), slice timing, head motion correction, spatial normalization (by using T1 image unified segmentation [29], normalized to Montreal Neurological Institute (MNI) space, resampled to 3 mm × 3 mm × 3 mm), spatial smooth (smoothed with 4 mm full-width at half-maximum Gaussian kernel), linear drift trends removal, nuisance covariates regression (including head motion parameters, global mean signal, white matter signal, and cerebrospinal fluid signal), and temporal filter (0.01–0.1 Hz).

### 2.3. Respiratory Motion Analysis

**Figure 1:**
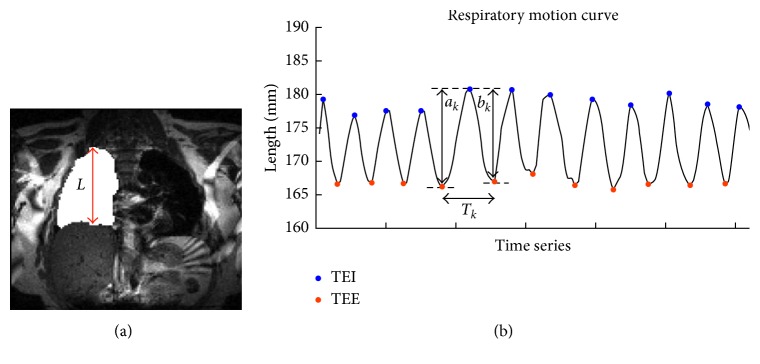
Characteristics of respiratory motion. (a) MRI image of thoracic-abdomen section in coronal view. *L* indicates respiration length, the distance from thoracic diaphragm to the top of the lung. (b) Sample of respiration curve. TEI = tail end of inspiration; TEE = tail end of expiration.

The MRI image of thoracic-abdomen section in coronal view was used to extract respiration data. The distance from thoracic diaphragm to the top of the lung was defined as respiration length (Figure1(a)). The influence of cardiac motion was regressed out by frequency depression of 1 Hz. The amplitude fluctuated during respiratory motion, which formed a respiratory motion curve (Figure1(b)). To evaluate the characteristics of respiratory motion, amplitude and tail-end of respiration were identified. For each volunteer, these indicators were calculated for both NS and HS to analyze interstate differences of respiratory motion.

#### 2.3.1. Respiration Amplitude

Respiration amplitude evaluates the variation of respiration length. As demonstrated in Figure1(b), in a single cycle, the amplitude is the averaged value from the peak to its two adjacent troughs (*a*_*k*_ and *b*_*k*_). For each volunteer, the respiration amplitude is defined as the weighted average of the amplitudes of all cycles, as follows:

(1) $$\begin{array}{c} A=\overset{K}{\underset{k=1}{\sum }}w_{k}\cdot \frac{a_{k}+b_{k}}{2}, \end{array}$$

where *A* is the averaged respiration amplitude of the volunteer, *K* is the total number of respiratory cycle, *w*_*k*_ is the weight of *k*th cycle to the entire respiration curve, and ∑_*k*=1_^*K*^*w*_*k*_=1.

In addition, we define amplitude deduction AD as follows:

(2) $$\begin{array}{c} \text{AD}=-\frac{A^{\text{HS}}-A^{\text{NS}}}{A^{\text{NS}}}\times 100\%, \end{array}$$

where *A*^NS^ indicates the amplitude in NS, *A*^HS^ indicates the amplitude in HS, positive AD represents decreased amplitude in HS, and negative AD represents increased amplitude in HS.

#### 2.3.2. Tail End of Respiration

Tail end of respiration includes tail end of inspiration (TEI) and tail end of expiration (TEE), corresponding to the peak and trough in the respiratory motion curve (Figure1(b)). For each volunteer, TEI/TEE averages all of the peaks/troughs.

(3) $$\begin{array}{c} \text{TEI}=\frac{1}{K}\overset{K}{\underset{k=1}{\sum }}P_{k},\\ \text{TEE}=\frac{1}{K}\overset{K}{\underset{k=1}{\sum }}Q_{k}, \end{array}$$

where *P*_*k*_ is the peak position of *k*th cycle and *Q*_*k*_ is the trough position of *k*th cycle.

### 2.4. Analysis of Spontaneous Brain Activity

To analyze the spontaneous brain activity in NS and HS, three voxel-wise measurements were calculated: fractional amplitude of low frequency fluctuation (fALFF), regional homogeneity (ReHo), and degree centrality (DC). Low frequency fluctuation is thought to reflect spontaneous brain activity [30,31]. Fractional ALFF is defined as the ratio of the power of the low frequency band (0.01–0.1 Hz) to the power of the entire detectable (0–0.25 Hz) frequency band [26]. ReHo calculates Kendall's coefficient concordance to evaluate the local signal synchronization by analyzing the similarity of time series of the chosen voxel with its neighboring voxels [27]. In this paper, ReHo was calculated by the synchronization of a voxel with its 26 neighboring voxels. DC is a voxel-wise measurement to estimate the global functional connectivity density between a voxel with all other voxels within the mask [28]. In this study, DC was calculated by summing up the number of voxels whose correlation coefficient with the target voxel reached a given threshold (*r* = 0.25). Additionally, the fALFF/ReHo/DC value of each voxel was converted to the *Z*-value by Fisher's Z transformation (through subtracting the global-brain mean value and then dividing by the global standard deviation) for standardization.

### 2.5. Statistical and Correlative Analysis

For the respiratory motion analysis, individual level and group level of interstate differences were identified by a two sample *t*-test (*p* < 0.05) and paired *t*-test (*p* < 0.05), respectively. Interstate difference of neural activity was identified by a paired *t*-test (*p* < 0.005, AlphaSim multiple comparison correction) within a grey matter mask on fALFF/ReHo/DC maps of two states. Afterwards, the clusters showing significant difference were taken as regions of interest (ROIs) for the Pearson correlative analysis between respiratory motion (amplitude, TEI, TEE) and neural activity. ROI signals of fALFF/ReHo/DC were extracted by averaging all of the within-ROI voxels. Moreover, to examine the correlation between neural activity and respiratory motion comprehensively, voxel-wise correlation within the grey matter mask of two states was calculated and compared, regressing out the covariates of demographic characteristics in Table1. For both NS and HS, the threshold of the correlation maps were set at *r* > 0.5, and the survival voxels of two states were combined as a mask to compare the interstate difference of neural correlation.

## 3. Results

### 3.1. Characteristics of Respiratory Motion

#### 3.1.1. Amplitude

**Figure 2:**
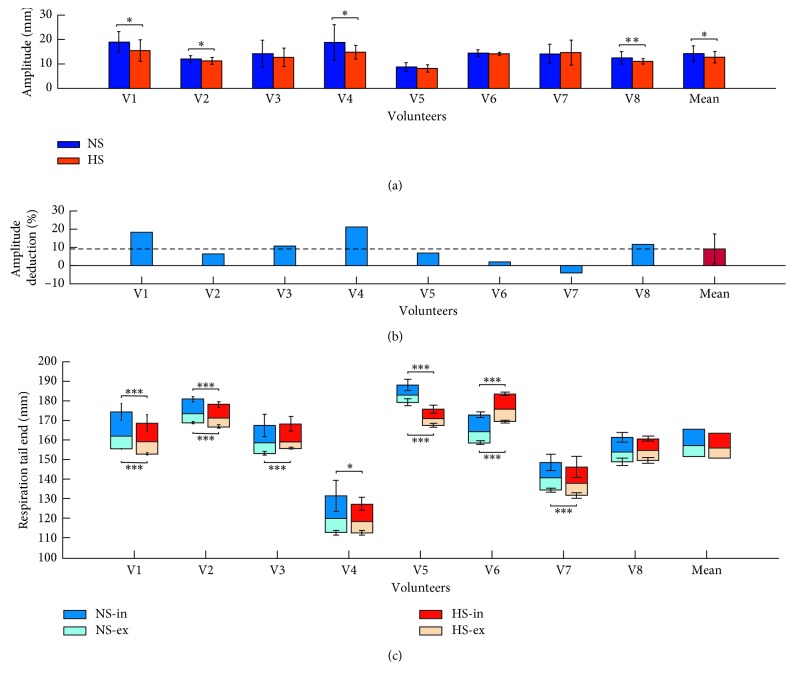
Amplitude and tail end of respiration in normal state (NS) and hypnosis state (HS). (a) Amplitudes. (b) Amplitude deduction in HS comparing to NS. (c) Tail end of inspiration (in) and expiration (ex). V1, V2, … , V8 were noted for individual volunteers. Statistical significance notation: ^∗^*p* < 0.05, ^∗∗^*p* < 0.005, and ^∗∗∗^*p* < 0.0005.

As demonstrated in Figure2(a), group level amplitude in NS was 14.23 ± 3.40 mm (mean ± SD) and in HS was 12.79 ± 2.49 mm. Significant lower amplitude was observed in HS in comparison with NS (*p*=0.0350). Seven out of eight volunteers were observed with reduced amplitude in HS, and the mean amplitude deduction was 9.2% (Figure2(b)). However, the unique one with increased mean amplitude in HS showed no significant (*p*=0.6394) higher values (V7 in Figure2(a)). These results indicated that hypnosis had an effect on respiratory control.

#### 3.1.2. Tail End of Respiration

Tail end of inspiration (TEI) and tail end of expiration (TEE) are the highest and the lowest respiration positions, respectively (Figure1(b)). In this paper, the boundary line (BL) between inspiration and expiration was defined as the averaged respiration amplitude of the entire respiratory motion curve. In HS, the mean TEI/BL/TEE across all volunteers was 163.45/155.81/150.65 mm, and all of them were lower than the results (165.49/156.89/151.26 mm) in NS (Figure2(c)). Although no significant interstate difference of TEI (*p*=0.4020), BL (*p*=0.6573), and TEE (*p*=0.7910) was observed in the group level, significant difference was demonstrated in individual volunteers (Figure2(c)).

### 3.2. Interstate Difference of Neural Activity

**Figure 3:**
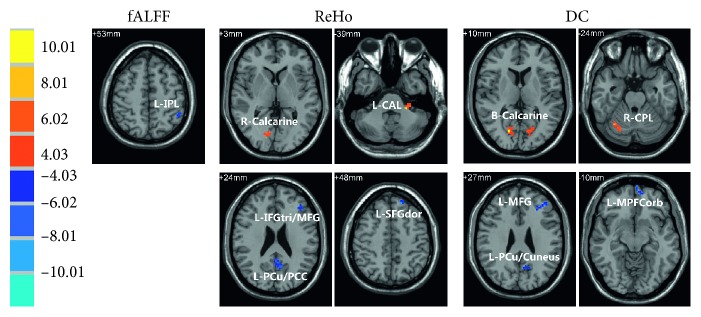
Statistical T-maps of interstate neural activity differences. The color-bar shows the statistical T-value for all maps. Red/blue indicates increased/decreased activity in hypnosis state, respectively. The threshold of T-maps are set at *p* < 0.005, AlphaSim-corrected, cluster size >324 mm^3^. fALFF = fractional amplitude of low frequency fluctuation; ReHo = regional homogeneity; DC = degree centrality; L = left hemisphere; R = right hemisphere; IPL = inferior parietal lobule; CAL = cerebellum anterior lobe; SFGdor = dorsolateral superior frontal gyrus; PCu = precuneus; PCC = posterior cingulate cortex; IFGtri = triangular part of inferior frontal gyrus; MFG = middle frontal gyrus; CPL = cerebellum posterior lobe; MPFCorb = medial orbital of prefrontal cortex.

The resultant statistical T-maps (voxel *p* < 0.005, AlphaSim-corrected without smoothness estimate, cluster size > 324 mm^3^, and grey matter mask) showed that there existed interstate difference in fALFF/ReHo/DC between NS and HS (Table2; Figure3). In HS, decreased fALFF was observed in the left inferior parietal lobule (IPL). As for ReHo, increased ReHo was observed in the left cerebellum anterior lobe (CAL) and right calcarine, while it was decreased in the left dorsolateral superior frontal gyrus (SFG), the left precuneus/posterior cingulate cortex (PCu/PCC), the left triangular part of inferior frontal gyrus, the and middle frontal gyrus (IFGtri/MFG). DC was increased in the bilateral calcarine and right cerebellum posterior lobe (CPL), whereas it was decreased in the left PCu/cuneus, left medial orbital of prefrontal cortex, and left MFG.

### 3.3. Correlative Analysis of Neural Activity and Respiratory Motion

**Figure 4:**
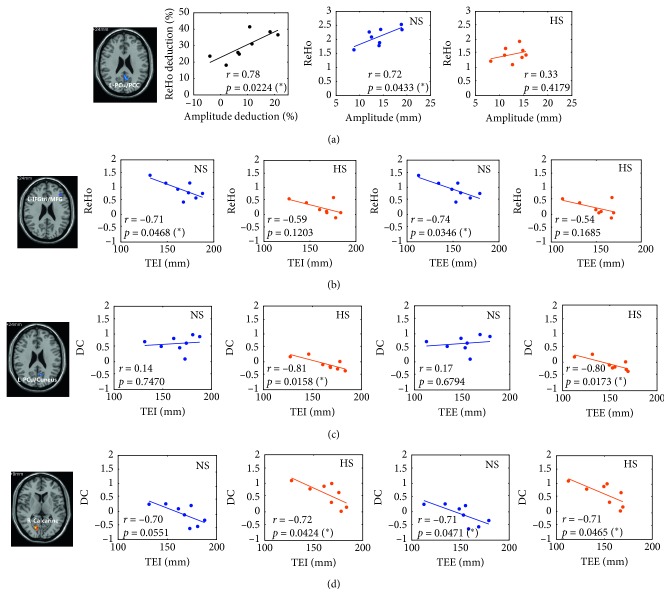
Correlation of neural activity and respiratory motion in brain regions showing interstate neural activity difference. (a) Correlation of ReHo and respiration in the left precuneus/posterior cingulate cortex (L-PCu/PCC). (b) Correlation of ReHo and respiration in the left triangular part of inferior frontal gyrus and middle frontal gyrus (L-IFGtri/MFG). (c) Correlation of DC and respiration in the left precuneus/cuneus (L-PCu/cuneus). (d) Correlation of DC and respiration in right calcarine. ReHo = regional homogeneity; DC = degree centrality; NS/HS = normal/hypnosis state; TEI/TEE = tail end of inspiration/expiration.

The significant correlations (*p* < 0.05) are demonstrated between brain activity (Table2; Figure3) and respiratory motion in Figure4. Positive correlation (*r*=0.78, *p*=0.024) was observed between ReHo deduction and amplitude deduction in the left PCu/PCC (Figure4(a)), while no significant correlation was observed between ReHo and amplitude in HS (*r*=0.72, *p*=0.0433 in NS; *r*=0.33, *p*=0.4179 in HS). Similarly, there was no significant correlation between ReHo and TEI/TEE in left the IFGtri/MFG (Figure4(b)). Oppositely, in HS, negative correlations between DC and TEI/TEE (*r*=−0.81, *p*=0.0158 for TEI; *r*=−0.80, *p*=0.0173 for TEE) were observed in the left PCu/cuneus, as compared with insignificant positive correlations in NS (Figure4(c)). Negative correlations of DC and TEI/TEE were both observed in the right calcarine in HS (Figure4(d)).

**Figure 5:**
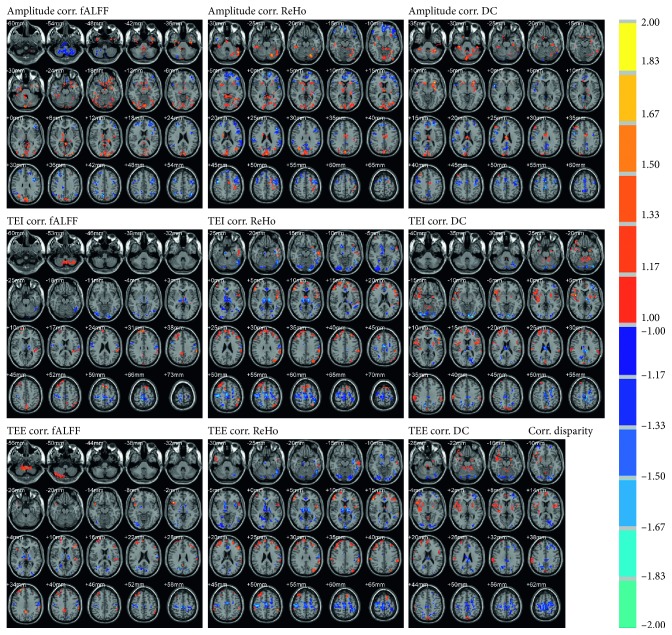
Interstate difference of voxel-wise correlation (corr.) between neural activity and respiratory motion, within combined mask of correlation maps of two states threshold at *r* > 0.5, and state-disparity of correlation coefficient over 1 and cluster size over 80 voxels (2160 mm^3^). The value of color-bar indicates coefficient in hypnosis state minus that in normal state. Red overlap indicates positive correlation in hypnosis state and negative in normal state, while blue indicates the negative correlation in hypnosis state and positive in normal state. TEI/TEE = tail end of inspiration/expiration; fALFF = fractional amplitude of low frequency fluctuation; ReHo = regional homogeneity; DC = degree centrality.

The voxel-wise correlation maps between neural activity and respiratory motion, within the combined mask of correlation maps (threshold at *r* > 0.5) of two states, were further threshold with state-disparity of correlation coefficient over 1 and cluster size over 80 voxels (2160 mm^3^). The state-disparity of correlation coefficient (the correlation coefficient in HS minus that of NS) over 1 accounted for opposite correlations of two states. The brain regions of the surviving voxels and the correlation coefficients are shown in Figure5 and Table3. In HS, positive correlations between amplitude and neural activity were observed in the CAL, middle cingulate cortex (MCC), cuneus, fusiform gyrus, and insula, and negative correlations were observed in the anterior cingulate cortex (ACC), prefrontal cortex (PFC), and precentral gyrus (PreC). However, these correlations of the corresponding brain regions were reversed in NS (Table3). The results of TEI and TEE were mostly consistent with each other. Different from NS, in HS, positive correlations between ReHo/DC and TEI/TEE were observed in the CAL, supramarginal gyrus (SMG), PFC, and insula, while negative correlations were observed in the CPL, MCC, supplementary motor area (SMA), PreC, postcentral gyrus (PostC), fusiform gyrus, and thalamus (Table3; Figure5).

## 4. Discussion

### 4.1. Hypnosis for Respiratory Control

In this study, hypnosis is intended to be applied for respiratory control without side effects in radiotherapy. Following hypnotic guidance with individual-customized content, volunteers feel peaceful and stay in a more stable and comfortable state of respiration. In our results, reduced amplitude of respiratory motion was observed in HS (Figures2(a) and 2(b)), which is consistent with our previous study [25]. Respiratory motion may induce fluctuation of dose distribution during radiotherapy, especially for lung and liver tumors. Tumor fluctuates in pace with respiratory motion. Therefore, reduction of respiratory amplitude is instrumental to suppress tumor motion, thus increasing dose rate for tumor target, meanwhile protecting organ at risk (OAR) from dose radiation. Moreover, as a clinical auxiliary method, hypnosis may help patients get into an inner peaceful state with less dependency on self-control during treatment, which is beneficial to treatment. These demonstrations suggest that hypnosis is an efficient alternative for respiratory control in radiotherapy.

In radiotherapy, planning target volume (PTV) covers the area of tumor motion, indicating that less scope and more stability of tumor motion are beneficial to more accurate PTV along with less dose. To accomplish this target, gating technology is recommended to be employed in radiotherapy by delivering radiation dose during the deepest of expiration in every respiratory period. Therefore, stable cycles and stability of TEE are critical for gating technology. However, given the prolonged treatment time of this technology and practical difficulties of operation, gating technology is not widely applied to radiotherapy. Instead, continuous dose radiation covering the area of tumor motion throughout the radiation-treatment procedure is a more stable and safe choice. In this case, reduced amplitude of respiration is crucial for radiotherapy. However, not all patients are adaptive to respiratory control by hypnosis. In this study, volunteers are all healthy; therefore, clinical trials of hypnosis are needed to examine its availability. Before it is applied to clinical treatment, sufficient pretrainings and evaluation of respiratory motion should be guaranteed to ensure its effectiveness and safety for the patient.

### 4.2. Neural Analysis of Hypnosis for Respiratory Control

Neural mechanisms of hypnosis have been explored for years, regarding its function of consciousness, cognitive processing, emotional regulation, attentional processing, executive control, and clinical stress/pain processing. Experimental results of interstate difference of neural activity demonstrated that alterations in HS were mostly located in the occipital cortex, cerebellum, and prefrontal cortex (Table2; Figure3). Moreover, results of ReHo and DC were consistent with each other. ReHo and DC are both measurements to evaluate signal synchronization or functional connectivity, where the former reflects regional synchronization and the later represents global synchronization.

In our results, activations were observed in the occipital cortex and cerebellum. The occipital cortex is known as the visual cortex, associated with visual processing. However, no visual-related task was induced during hypnosis experiment. A possible explanation is that it formed a picture in volunteers' minds when following hypnotic guidance. Increased neural activity in the cerebellum was also observed. The cerebellum involves in the function of motor control, perceptual processes, and sensory perception [32,33] and takes part in the charge of interoceptive processing [34] and emotional processing [35]. Moreover, the anterior lobe and posterior lobe associate with different functions, corresponding to sensorimotor CAL [33] and cognitive CPL [36]. In our results, neural activity in the anterior lobe and posterior lobe of the cerebellum were both demonstrated to be increased. The above results indicate that visual, sensorimotor, and cognitive processings are involved in hypnosis.

Contrary to the occipital cortex and cerebellum, decreased activity was demonstrated in the prefrontal cortex and PCu/PCC. The prefrontal cortex involves in complex cognitive behavior [37] and various subregions' response for different functions. Deactivations of the prefrontal cortex contained MPFC and dorsolateral prefrontal cortex (DLPFC). Both MPFC and PCu/PCC are critical parts of the default mode network (DMN), which activates in task-deprived state and deactivates in task-evoked state [38]. On the other hand, DLPFC is well known for its executive task-induced role in executive control processing [34] and attentional processing [39,40]. Contrary to our results, activation in MPFC has been observed in strong emotional arousal [41]. Consistently, disrupting DLPFC activity is observed in subjective response to hypnotic suggestion [42]. Decreased activity in both DMN regions and executive control regions may imply modulation of emotion and executive processing in hypnosis.

### 4.3. Neural Correlation of Respiratory Motion

A great number of studies have been working on the neural correlations between hypnosis and psychological performance. However, the neural correlations between hypnosis and physiology are rarely studied. In this study, we examined the correlation between neural activity and physiological performance (respiratory motion) during hypnosis. Within the brain regions showing significant interstate differences, significantly negative correlations were observed between DC and respiratory motion in the visual cortex in HS (Figures4(c) and 4(d)), while correlations were insignificant in PCu/PCC (Figure4(a)) and DLPFC (Figure4(b)). Activation in the visual cortex implies visual processing during HS; however, negative neural correlation of amplitude in visual cortex may indicate that less in-mind visual interruption helps for amplitude reduction. Insignificantly positive neural respiration in critical role of DMN (PCu/PCC), together with insignificantly negative results in critical role of executive control network (DLPFC), supports the breakout of the default brain state and arouse of executive brain in hypnosis for respiration control, which are identified through respiratory characteristics. Therefore, neural correlation of respiratory motion is an informative way to explore the potential mechanism of hypnosis for respiration control.

Although indicative correlation results showed interstate difference in some brain regions, identification of these regions was not so much convincing in terms of multiple comparison corrections which were carried out without smoothing estimation when clustering the statistical maps [43]. To make up this shortcoming, whole brain voxel-wise correlation was further examined. It was demonstrated that, in HS (opposite to the results of NS), positive correlations between neural activity and respiratory amplitude were observed in CAL, MCC, fusiform gyrus, and insula, while negative correlations were observed in ACC, PCu/PCC, prefrontal cortex, and sensorimotor area (PreC/PostC/SMA) (Figure5; Table3). Increased neural activity (Figure3; Table2) and positive- neural-respiratory correlation in the cerebellum, together with decreased neural activity (Figure3; Table2) and negative neural-respiratory correlation of amplitude in PCu/PCC, emphasize the consistent involvement of these regions during hypnosis for respiratory control. MCC and ACC are both cingulate regions associated with cognitive processing [44,45]. However, inconsistent results of their correlations of brain activity and respiratory motion may reveal their different neurophysiological functional roles in hypnosis. During hypnotic intervention, negative neural-respiratory correlations in sensorimotor areas (PreC/PostC/SMA) reveal the involvement of motor and sensory processing [46] during hypnosis for respiratory control. Interestingly, MPFC and DLPFC were observed with positive correlation between neural activity and TEE/TEI, while there was a negative correlation between neural activity and respiration amplitude (Figure5; Table3). The prefrontal cortex is suggested to be involved in the involuntariness in response to hypnotic suggestion [47]. These results further implicate that the prefrontal cortex plays a critical role during hypnosis for respiratory control.

Positive correlations were shown in both respiratory amplitude and TEE/TEI (Figure5; Table3). Studies observed with insula activation suggest that the insula is associated with awareness [48], self-representation, and emotional processing [49]. Additionally, the SMG, fusiform gyrus, and thalamus are robust brain areas that showed significant correlation between fALFF/ReHo/DC and TEE/TEI (Figure5; Table3). The SMG is part of Wernicke's area associated with semantic representation [50]. Positive correlation observed between brain activity in the SMG and respiratory motion during hypnotic intervention may result from hypnotic voice guidance from the hypnotist. The fusiform gyrus is a functionally defined region in visual face recognition [51,52]; however, this function is not relevant to our study. We hypothesized that the fusiform gyrus works together with the default mode network and executive control network during hypnotic intervention for respiratory control, similar to its role in facilitating social motivation with large-scale networks [53]. Hypnosis participates in the regulation of consciousness [13], and a clinical study suggests that lesions in thalamus may affect the level of consciousness [54]. Therefore, negative correlation results of the thalamus may indicate a kind of altered state of consciousness during hypnosis.

Hypnosis has been focused on its psychological aspect in many studies, whereas we highlight its physiological effects of respiratory control in this study. As a psychological intervention, hypnosis is not only for respiratory control, but also attenuates the pain of patients during radiotherapy. Our results suggest the involvement of cognitive processing, emotional regulation, sensorimotor processing, and executive control processing in hypnosis for respiratory control. Though significant results are observed, however, small sample size and individual specificity of hypnotic contents may miss other potential relations. Therefore, adequate patient cases are needed for further understanding the neural and molecular mechanisms of hypnosis for respiration control.

## 5. Conclusion

In conclusion, this study examined the effect of hypnosis on respiration control and investigated spontaneous brain activity by measuring fALFF/ReHo/DC as well as the correlation between neural activity and respiratory motion. Reduced respiratory motion amplitude and stable respiratory cycle were observed in hypnosis with relaxation suggestion. Increased brain activity was observed in the visual cortex and cerebellum, while it was decreased in the prefrontal cortex and PCu/PCC. Positive neural correlations of respiratory amplitude were shown in the anterior lobe and insula, while they were negative in the prefrontal cortex and sensorimotor areas. These findings reveal the involvement of cognitive, executive control, and sensorimotor processing in hypnosis for respiratory control.

## Figures and Tables

**Table 1:** Demographic characteristics of eight healthy volunteers.

	Mean ± SD	Range
Age (year)	33 ± 9	23–47
Gender (female/male)	6/2	/
Height (cm)	166 ± 12	155–185
Weight (kg)	66 ± 21	52–105

SD = standard deviation.

**Table 2:** Clusters showing interstate fALFF/ReHo/DC difference.

Brain region	Brodmann area	Cluster size (mm^3^)	Peak MNI coordinates	Peak T value
*Interstate fALFF difference*				
L-IPL	40	324	−48 −45 42	−5.65
*Interstate ReHo difference*				
L-CAL	/	324	−24 −36 −39	10.73
R-calcarine	18	459	15 −78 3	6.56
L-SFGdor	9	378	−15 42 48	−8.80
L-PCu/PCC	23/30	702	0 −51 21	−7.39
L-IFGtri/MFG	45/46	351	−39 30 27	−6.46
*Interstate DC difference*				
R-calcarine	18/17	1566	18 −75 9	12.14
R-CPL	/	486	33 −63 −24	8.36
L-calcarine	17	891	−15 −75 12	7.47
L-PCu/cuneus	23	378	−9 −63 24	−7.37
L-MPFCorb	11	351	−3 63 −12	−7.11
L-MFG	46	432	−39 39 30	−6.90

fALFF = fractional amplitude of low frequency fluctuation; ReHo = regional homogeneity; DC = degree centrality; MNI = Montreal Neurological Institute; L = left hemisphere; R = right hemisphere; IPL = inferior parietal lobule; CAL = cerebellum anterior lobe; SFGdor = dorsolateral superior frontal gyrus; PCu = precuneus; PCC = posterior cingulate cortex; IFGtri = triangular part of inferior frontal gyrus; MFG = middle frontal gyrus; CPL = cerebellum posterior lobe; MPFCorb = medial orbital of prefrontal cortex. Positive/negative T-value indicates increased/decreased activity in hypnosis state.

**Table 3:** Brain regions showing significant interstate difference of voxel-wise correlation between neural activity and respiratory motion.

	fALFF				ReHo				DC			
	Brain regions	Voxels	NS-r	HS-r	Brain regions	Voxels	NS-r	HS-r	Brain regions	Voxels	NS-r	HS-r
Amplitude	B-CAL	103	−0.62	0.59	B-MCC	155	−0.64	0.54	B-CAL/fusiform	250	−0.52	0.66
	B-fusiform	319	−0.64	0.61	B-cuneus/fusiform	1037	−0.56	0.66	B-MCC	198	−0.61	0.61
	B-cuneus	1064	−0.63	0.60	L-CAL	113	−0.60	0.61	B-cuneus	213	−0.64	0.58
	B-CPL	404	0.47	−0.70	L-insula	99	−0.53	0.67	L-insula/Put	215	−0.68	0.61
	B-MFG/SFG	533	0.60	−0.64	R-caudate/thalamus	242	−0.60	0.59	R-caudate	98	−0.63	0.56
	B-PreC	235	0.65	−0.59	B-MPFC/ACC	599	0.60	−0.66	B-SMA/PreC/PostC	945	0.67	−0.58
	B-IPL	285	0.65	−0.56	B-SMG	188	0.58	−0.59	B-ACC	102	0.57	−0.67
					L-PreC	709	0.59	−0.64	B-PCu/PCC	221	0.51	−0.65
TEI	B-CAL	124	−0.70	0.47	B-PFC	1052	−0.64	0.55	B-CAL	139	−0.53	0.59
	R-MFG/SFG	265	−0.64	0.55	L-SMG	159	−0.66	0.62	B-SMG	195	−0.61	0.59
	B-PCu/PCC	150	−0.57	0.62	L-insula	124	−0.63	0.56	B-PFC	248	−0.75	0.42
	L-SMG	219	−0.62	0.60	B-thalamus	485	0.63	−0.65	B-insula/Put	887	−0.56	0.65
	B-fusiform	205	0.66	−0.53	B-fusiform	494	0.66	−0.56	B-cuneus	102	−0.54	0.61
	B-thalamus	160	0.70	−0.53	B-ACC	112	0.51	−0.71	R-fusiform/CPL	525	0.60	−0.60
	B-SMA/PostC/PreC	534	0.56	−0.68	B-MCC/PostC/PreC	1249	0.61	−0.66	B-SMA/PreC/MCC	1325	0.58	−0.65
					B-LG	316	0.62	−0.58	R-PCu	128	0.62	−0.59
TEE	B-CPL/CAL	241	−0.70	0.50	B-PFG	881	−0.63	0.57	B-CAL	191	−0.57	0.55
	B-PFC	323	−0.64	0.54	B-SMG	207	−0.65	0.58	B-amygdala/Put/insula	855	−0.56	0.65
	L-IPL	144	−0.64	0.59	B-thalamus	439	0.66	−0.61	R-SMG	115	−0.63	0.54
	B-PCu/PCC	85	−0.69	0.58	B-PostC/PreC	1201	0.65	−0.60	B-MPFC/R-MFG	169	−0.73	0.44
	R-insula	97	−0.58	0.68	B-ACC	104	0.60	−0.63	B-CPL	514	0.64	−0.56
	B-SMA/PostC/PreC	412	0.58	−0.64	B-CPL/fusiform	402	0.66	−0.52	B-SMA/PreC/MCC	1443	0.60	−0.64
	B-thalamus	167	0.67	−0.54	B-LG	214	0.68	−0.49	L-MFG	221	0.66	−0.55
	R-fusiform	105	0.71	−0.50								

Paired *t*-test of correlation coefficients of two states across voxels show significant interstate difference (*p* < 0.000001) for every cluster. TEI/TEE = tail end of inspiration/expiration; fALFF = fractional amplitude of low frequency fluctuation; ReHo = regional homogeneity; DC = degree centrality; NS-r/HS-r = correlation coefficient in normal/hypnosis state; L/R/B = left/right/bilateral; LG = lingual gyrus; PCu = precuneus; CAL/CPL = cerebellum anterior/posterior lobe; ACC/MCC/PCC = anterior/middle/posterior cingulate cortex; MPFC/PFC = (medial) prefrontal cortex; SFG/MFG/IFG = superior/middle/inferior frontal gyrus; PreC/PostC = precentral/postcentral gyrus; SMA = supplementary motor area; SMG = supramarginal gyrus; IPL = inferior parietal lobule; Put = putamen; fusiform = fusiform gyrus.
